# Virus-Induced Tumorigenesis and IFN System

**DOI:** 10.3390/biology10100994

**Published:** 2021-10-01

**Authors:** Marco Iuliano, Giorgio Mangino, Maria Vincenza Chiantore, Paola Di Bonito, Paolo Rosa, Elisabetta Affabris, Giovanna Romeo

**Affiliations:** 1 Department of Medico-Surgical Sciences and Biotechnologies, Sapienza University of Rome—Polo Pontino, 04100 Latina, Italy; marco.iuliano@uniroma1.it (M.I.); giorgio.mangino@uniroma1.it (G.M.); p.rosa@uniroma1.it (P.R.); 2 Department of Infectious Diseases, Istituto Superiore di Sanità, 00161 Rome, Italy; mariavincenza.chiantore@iss.it (M.V.C.); paola.dibonito@iss.it (P.D.B.); 3 Department of Science, Roma Tre University, 00146 Rome, Italy; elisabetta.affabris@uniroma3.it

**Keywords:** oncogenic viruses, IFNs, tumor immune surveillance, microRNAs, extracellular vesicles

## Abstract

**Simple Summary:**

This review aims to collect recent studies on the complex relationship between the host innate response to oncogenic viruses (i.e., HPV, HTLV-1, MCPyV, JCPyV, Herpesviruses, HBV, HCV) and tumorigenic processes by focusing mainly on regulatory crosstalks between viral components and the type I IFN system. It is a picture of new mechanisms by which type I IFNs may be affected and, in turn, affect signaling pathways to mediate anti-proliferative and antiviral responses in virus-induced tumorigenic context. Studies on cellular and viral miRNAs machinery, as well as cellular communication and microenvironment modification via classical secretion mechanisms and extracellular vesicle-mediated delivery are described.

**Abstract:**

Oncogenic viruses favor the development of tumors in mammals by persistent infection and specific cellular pathways modifications by deregulating cell proliferation and inhibiting apoptosis. They counteract the cellular antiviral defense through viral proteins as well as specific cellular effectors involved in virus-induced tumorigenesis. Type I interferons (IFNs) are a family of cytokines critical not only for viral interference but also for their broad range of properties that go beyond the antiviral action. In fact, they can inhibit cell proliferation and modulate differentiation, apoptosis, and migration. However, their principal role is to regulate the development and activity of most effector cells of the innate and adaptive immune responses. Various are the mechanisms by which IFNs exert their effects on immune cells. They can act directly, through IFN receptor triggering, or indirectly by the induction of chemokines, the secretion of further cytokines, or by the stimulation of cells useful for the activation of particular immune cells. All the properties of IFNs are crucial in the host defense against viruses and bacteria, as well as in the immune surveillance against tumors. IFNs may be affected by and, in turn, affect signaling pathways to mediate anti-proliferative and antiviral responses in virus-induced tumorigenic context. New data on cellular and viral microRNAs (miRNAs) machinery, as well as cellular communication and microenvironment modification via classical secretion mechanisms and extracellular vesicles-mediated delivery are reported. Recent research is reviewed on the tumorigenesis induced by specific viruses with RNA or DNA genome, belonging to different families (i.e., HPV, HTLV-1, MCPyV, JCPyV, Herpesviruses, HBV, HCV) and the IFN system involvement.

## 1. Introduction

Tumorigenesis produced by oncogenic virus infection appears to be the result of a fine combination of pro- and anti-viral factors, i.e., interferons (IFNs) and IFN-stimulated genes (ISGs).

Type I IFNs are cytokines of early response to viral infection with a profound effect on cell growth. IFN anticancer activity appears to be based on direct and indirect mechanisms leading to decreased expression of viral oncogenes, increased expression of tumor suppressor genes, modification of cell cycle progression, induction of apoptosis, and senescence [1]. Numerous stimuli that initiate senescence are cancer-related stresses. Since senescent cells counteract oncogenic transformation, it has been proposed that senescence can fight tumorigenesis. Inactivation of pRb-p53 signaling in proliferating cells is able to prevent cellular senescence. On the other hand, chronic activity of these tumor suppressors in response to non-physiological conditions of tissue culture or to oncogenes favors growth arrest [2].

More recently, another mechanism through which IFNs exert their antiviral action appears to be the modulation of microRNA (miRNA) expression. MiRNAs are small, non-coding, highly conserved RNAs able to arrest target mRNA translation by a post-transcriptional regulation. Moreover, in the case of oncogenic viruses, the dysregulation of miRNA activity due to IFNs could result in anticancer effects. However, miRNAs themselves can affect IFN expression [3]. The expression of many miRNAs is dysregulated in different cancers; oncogenic miRNAs or oncosuppressor miRNAs modulation is responsible for the control of cell proliferation, invasion, and metastasis [4]. Furthermore, it has been reported that infected cells can modify the release as well as the content of extracellular vesicles (EVs). EVs are small bilayer lipid membrane vesicles, released by all cells as delivery vehicles involved in cell-to-cell communication, classified according to their size and origin. Viral proteins, nucleic acids, miRNAs, and even entire virions can be packaged into EVs. EVs derived from infected cells are garnering increasing attention due to their abilities to participate in intercellular communication or transfer bioactive factors between virus-infected cells and neighboring normal cells possibly modifying the microenvironment affecting tumor development and immune resistance [5].

Here, we review recent researches on the tumorigenesis induced by specific viruses, with RNA or DNA genome, belonging to different families (i.e., HPV, HTLV-1, MCPyV, JCPyV, Herpesviruses, HBV, HCV) and the IFN system involvement. The review aims to describe a picture of new mechanisms by which IFNs may be affected and, in turn, affect signaling pathways to mediate anti-proliferative and antiviral responses in a virus-induced tumorigenic context.

## 2. IFN System

Human type I IFNs consist of a family of IFN proteins encoded by at least 13 IFNα subtype genes (i.e., IFN-α1, -α2, -α4, -α5, -α6, -α7, -α8, -α10, -α13, -α14, -α16, -α17 and -α21), one IFN-β gene, one IFN-ε, one IFN-κ gene, and IFN-ω gene, whereas Type III IFNs are encoded by three closely positioned genes on human chromosome 19 (i.e., IFN-λ1, -λ2, -λ3). They represent the first line of defense against viral infection in the innate immune response [6]. Their production and release at the cellular level occur after the detection of viral elements by innate immune sensors, activation of downstream signaling pathways, and transcriptional events. Downstream events starting from the specific involved cellular receptor and leading to the transcription of hundreds of ISGs are able to code for multiple proteins committed to “interfere” with viral replication phases [7]. Type I and type III IFNs also activate the adaptive immune responses against viruses. Depending on the context in which IFN signaling is induced, the IFN response may result in a protective or pathogenic action against viruses.

Interestingly, numerous viruses that cause both acute and persistent infections, as well as oncogenic viruses, evolved to evade or contrast the IFN action in the host by acting with specific viral proteins able to alter IFN signaling at different stages.

**Figure 1:**
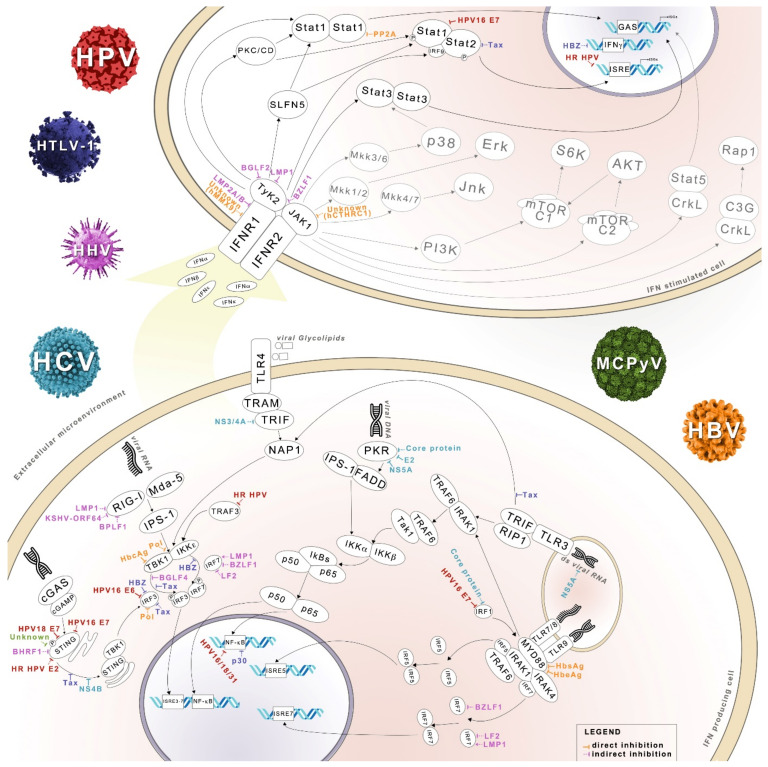
Signal transduction pathway network relevant for type I IFNs production and response to IFNs stimulus. Summary of the interfering oncoviruses proteins of different colors label is reported: HPV proteins (red), HTLV-1 (purple), HHV (pink), HCV (light blue), MCPyV (green), and HBV (orange). Faded pathways refer to non-canonical pathways involved in type I IFN signaling [8].

Type I IFNs, acting in both autocrine and paracrine mode, generate an antiviral state supported by the rapid expression of ISGs [7], (Figure 1).

This antiviral defense also implies the recruitment and activation of different immune cells and IFN-induced proinflammatory cytokines to promote virus clearance via a well-controlled and sustained adaptive immune response [9,10]. The production of IFN is initiated by sensing of viral RNA by cellular PRRs, including RIG-I, MDA5, and LGP2 along with the cooperation of MDA5 and the downstream adaptor MAVS [11,12]. Viral RNA can be also detected when located in the endosomal compartment by the TLRs. TLR3 detects double-stranded RNA, TLR7 and TLR8 detect single-stranded RNA. Innate signaling cascade includes the downstream adaptor protein molecules for TLRs, MyD88 (for TLR4, TLR7, TLR8), and TRIF (for TLR3, TLR4) [13]. The phosphorylation of the IFN gene “master regulators”, IRF3 and IRF7 through TRAF3, TBK1 and IKKε [14], and the consequent dimerization and translocation of IRF3 and/or IRF7 into the nucleus, induce the expression (and release) of IFN-I as well as of a subset of early ISGs [15]. In addition, NF-κB induces the expression of pro-inflammatory cytokines (e.g., IL-1, IL-6, TNF-α). The released IFN-I binds to the interferon-α and -β receptor (IFNAR, composed of the IFNAR1 and IFNAR2 subunits) on the surface of target cells. This binding leads to the activation of Jak tyrosine kinases, Tyk2, and JAK1, and to the phosphorylation of specific tyrosine residues of the receptor chains. The modified domains of the IFNAR serve as docking sites for the recruitment of STATs [16]. Phosphorylated STAT1 and STAT2 heterodimerize and associate the DNA binding protein IRF9 to form a complex named ISGF3. ISGF3 translocates to the nucleus and binds an ISRE DNA to activate ISGs transcription, thus inducing the expression of numerous ISG products that establish the antiviral state [17,18]. Likewise, viral RNA may also be recognized by its double-stranded RNA elements using the cellular PKR. This ISG enzyme, under IFN-I influence, inactivates the initiation factor eIF2α to block protein synthesis in infected cells [19].

## 3. IFN Signaling and MicroRNA

In the last few years, the involvement of specific cellular and viral miRNAs related to type I IFNs has been reported. Oncogenic viruses activate miRNA-mediated mechanisms producing specific cellular pathways modifications, thus favoring tumor development. Specific miRNAs are frequently located in virus integration sites or involved in the initiation and progression of virus-induced human cancer. It has been recently suggested that miRNAs present in the cells may participate in host-virus interactions, influencing viral replication [20,21,22]. A subset of host miRNAs appears to be specifically regulated by oncogenic virus infection. The elevated or decreased expression of numerous miRNAs has been attributed to various viral oncoproteins responsible for cell immortalization and transformation. Besides the ability to deregulate cellular miRNAs, many mammalian viruses have evolved strategies to counteract cellular antiviral defense by encoding their own miRNAs thus favoring virus-induced tumorigenesis through the deregulation of key elements of type I IFN system (see Table 1 at the end of the next paragraph).

Early research on IFNs led to the discovery of the JAK-STAT pathways. More recently, additional evidence has established the involvement of other receptor-activated signaling pathways in the activities of type I IFNs (i.e., MAPK, mTOR, and PKC pathways) [23], as well as of specific miRNAs that appear to play a significant role in the regulation of IFN-signaling responses. It has been shown that the direct regulation of STAT activity by miRNAs affects specific gene expression, by modifying the consequent cytokine-inducible events [24] as well as the critical involvement of specifically regulated STAT activity in a variety of cell processes. JAK-STAT signaling is affected by various miRNAs that target SOCS proteins [25,26,27]. It is important to highlight that IFNs are able to regulate miRNAs at the level of the miRNA biogenesis. In fact, IFNs appear to be able to affect the endoribonuclease Dicer expression [28] as well as Dicer expression, which appears to be necessary for correct IFN expression and antiviral activity [29], indicating that the interplay between IFNs, virus infection, and miRNAs involves also the miRNA biogenesis machinery. Therefore, even if during the years the IFN research has been mainly focused on the induction of interferon expression and on interferon-induced proteins, new evidence indicates that the IFN system may also have a role in the control of the expression of a number of non-coding RNA genes, especially miRNAs. This suggests that IFNs, ISGs, and miRNAs act synergistically to induce a potent cellular environment non-permissive for virus replication. On the other hand, specific miRNAs may act as critical regulators of IFNs expression [30,31] as well as of ISG [32,33,34]. Altogether, this system may be included in the cellular innate response to virus infection and virus-induced cancer.

It has been reported that infected cells release EVs thus modifying the microenvironment, affecting tumor development and immune resistance [5]. In this respect, we and other authors have studied the effects of viral oncoproteins in influencing cell microenvironment by classical secretion and extracellular vesicles delivery of inflammatory mediators as well as of specific miRNAs [35,36,37]. Considering the aforementioned interplay between IFNs and miRNAs, the possibility that oncogenic viruses infected cells can emit miRNAs through EVs suggests that this delivery can be an efficient way to interfere with the host immune response.

## 4. IFN System and Oncoviruses

### 4.1. HPV

Human papillomaviruses (HPVs) are double-stranded DNA viruses with icosahedral capsid structure that infect basal keratinocytes of the mucosal and cutaneous epithelia. A small group of HPV genotypes are defined as high risk (HR) because their infection can cause different types of tumors, among which cervical cancer is the most represented in women worldwide. The HPV type 16 is the causative agent of 61% of all cervical cancers and is also involved in other anogenital and head and neck cancers both in men and in women [38].

HPV E6 and E7 are the viral oncoproteins with the most important role in promoting cell transformation and tumorigenesis. By interfering with different molecular pathways, these oncoproteins can drive cells toward malignancy through the induction of all the hallmarks of cancer. The key function of HPV E6 and E7 is exerted by targeting and inhibiting the oncosuppressors p53 and pRb, respectively. However, these viral oncoproteins are able to impair the function of many other cellular proteins as well as affect microRNAs expression with less known mechanisms [36,39].

The immune system of the majority of people is able to clear HPV infection within 1–2 years. However, in some individuals, HPV infection persists thus increasing the risk for tumor progression. It has been reported that HPV interferes with the functions of the host immune system, leading to the failure of virus clearance and the consequent viral persistence [40,41]. However, it is not yet fully understood how HPV evades immune response. Escape from the immune system can be a consequence of the “hidden” HPV life cycle that takes place within the boundary of the lamina basalis, distant from the dermal immune cells surveillance. In addition, the immune system is not alerted by HPV infection since it does not cause viremia, cell death, or cell lysis required for a rapid inflammatory response.

Keratinocytes can produce type I IFNs and pro-inflammatory cytokines upon PRR ligation caused by viral infection, but HR-HPV types affect PRR- and type I IFN-induced signaling pathways. In particular, HPV-16, -18, and -31 downregulate TLR3-induced cytokine expression, and ISGs expression [42,43,44].

It is well established that the expression of the early oncogene E6 and E7 by HR-HPVs is required for interfering with the immune system response. E6 and E7 oncoproteins of HR-HPVs are tumor-specific antigens expressed in precursor lesions and in tumors. These proteins exert a pivotal role in viral immune evasion since they interact with multiple cellular proteins thus promoting tumor development.

A central component of the innate immune system to sense the presence of cytosolic DNA and trigger the activation of defense mechanism through IFN pathways is the cGAS/STING pathway [45,46]. Responses mediated by type I IFNs mainly affect proliferation and apoptosis activities, thus inhibiting viral persistence. As a matter of fact, to support persistent infection, HPV oncoproteins can counteract the cGAS/STING/IRF3 axis as well as the activation of ISG responses [47,48,49,50,51].

HR-HPV E7 inhibits STING and STING-dependent IFN-I responses since it has been shown that the increased expression of this oncoprotein is correlated to decreased levels of STING protein and decreased phosphorylation of TBK1. However, E7 oncoproteins of different HR-HPV genotypes interfere with cGAS/STING pathway with various mechanisms. HPV18 E7 interacts with the STING complex whereas HPV16 E7 colocalizes with NLRX1a, PRR members, and mitochondria, thus destabilizing STING. NLRX1 can control IFN-I production mediated by cytoplasmic PRRs such as STING [52,53,54], allowing an indirect suppression of IFN-I by HPV16 E7.

Another HR-HPV early protein, E2, downregulates the expression of STING and IFNκ [55], whose levels are also reduced by HR-HPV E6 [55,56]. The suppression of IFNκ expression inhibits ISGs transcription and could play a pivotal role in the promotion of tumors induced by HPVs.

HPV-16 E6 oncoprotein binds to IRF3 thus inhibiting its transcriptional activity [48] and HPV-16 E7 suppresses IFNβ transcription by binding to IRF1 and recruiting histone deacetylases (HDACs) to the IFNβ promotor site [57,58]. It has been reported that HR-HPV infection leads to the inhibition of IRF signaling through the upregulation of the endogenous deubiquitinase ubiquitin carboxy-terminal hydrolase L1 (UCHL1) and the consequent deubiquitination of K63-linked poly-ubiquitin chains from TRAF3. This results in reduced TBK1-TRAF3 interaction, IRF3 phosphorylation, and IFNβ expression [59].

**Figure 2:**
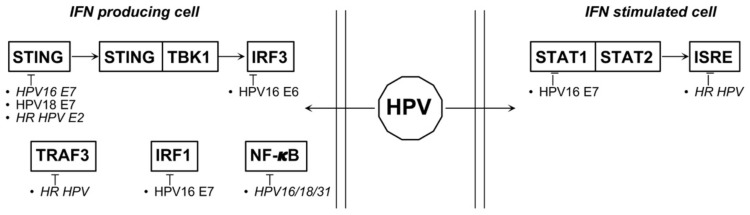
Proteins and miRNAs able to deregulate type I IFNs relevant pathways during HPV associated tumorigenesis. Italics represent indirect action.

HPV-16 E7 prevents IRF1 expression through the suppression of IFNγ-induced STAT1-Tyr701 phosphorylation and the inhibition of IFNγ-mediated upregulation of MHC-I expression via the JAK1/JAK2/STAT1/IRF-1 signal transduction pathway [60]. The overexpression of the yes-associated protein 1 (YAP1) oncogene in cervical epithelial cells can induce the downregulation of genes encoding ISGs that directly inhibit virus infection, such as MX1, ISG15, APOBEC3G, OAS1, TRIM5, and IFI44L. Thus, YAP1 can counteract host cell immune response and promote HPV-induced tumorigenesis [60] (HPV effects on type I IFN system are summarized in Figure 2).

During the years, many efforts have been made to understand whether IFNs could be employed to treat HPV-induced lesions and tumors. Controversial results are probably due to the multiple abilities of the proteins of the different HPV genotypes to interfere with IFN pathway. More recently, the growing knowledge of the interplay between miRNAs and IFNs has been studied also in the context of HPV infection. Therefore, the study of the role of IFN in the expression of miRNAs modulated by HPV oncoproteins is of particular interest. We and others have reported that E6 and E7 viral oncoproteins are able to modulate miRNAs with anti- or pro-tumorigenic properties [36,61]. We have shown that IFNβ is able to counteract the effect of HPV-16 E6 and E7 oncoproteins on the expression of miR-10a, -18a, -19a, -21, -34a, -98, -182, -194, -590-5p. Of note, IFN-β induces apoptosis in the same cell system independently of p53 [62]. Another study reported that IFN-β up-regulation of miR-129-5p leads to the reduction of the expression of HR-HPV18 E6 and E7 oncogenes [63]. On the other hand, miRNAs can be involved in the host-virus response to HPV by activating IFN signaling following virus infection. It has been shown that miR-122 could decrease HPV16 E6 mRNA expression, by directly targeting E6 mRNA, and it could inhibit SOCS1, thus inducing IFN-I production and leading to the activation of down-stream responses in signaling pathway [64]. MiR-221 could be able to inhibit HPV 16 E1-E2 mediated DNA replication by targeting SOCS and inducing Type I IFN response [65].

Modulated miRNAs can be delivered by infected cells to the microenvironment and to the surrounding cells through the release of EVs. We and others have reported that HPV E6 and E7 expressing keratinocytes can promote tumorigenesis by EV delivery of significant miRNAs as well as HPV oncogenes [35,36,66].

Therefore, the characterization of the complex interactions among IFNs, miRNA pathways, and EV delivery in HPV expressing cells could be of particular interest in the design of biomolecular strategies in the IFN therapy of lesions and tumors induced by HPV infection.

### 4.2. HTLV-1

The human T-lymphotropic virus type 1 (HTLV-1) is the first oncogenic member of the Retroviridae family, Deltaretrovirus genus [67,68], being the causative agent of the adult T-cell leukemia/lymphoma (ATLL). In a smaller percentage of subjects, HTLV-1 causes an inflammatory neurodegenerative disease defined as HTLV-1 associated myelopathy/tropical spastic paraparesis (HAM/TSP) [69,70]. In the latter case, the etiological agent could also be another member of the family, HTLV-2, unable to induce leukemia. HTLV-1 induces oncogenesis through the induction of multiple mechanisms as chronic inflammation, activation of oncogenic pathways, inhibition of oncosuppressors, apoptosis, DNA damage repair, and autophagy. Transcriptional activator x (Tax) and basic leucine zipper factor (HBZ) proteins play a pivotal role in these functions: both proteins induce hTERT at least in resting T-cells; Tax-1 inhibits the nucleotide excision repair (NER) and interferes with the function of p53. On the other hand, HBZ interferes with the antiproliferative activity of C/EBPα and ATF3, suppresses the canonical Wnt pathway, and inhibits apoptosis and autophagy in HTLV-1 infected cells (for a detailed review see [71]).

**Figure 3:**
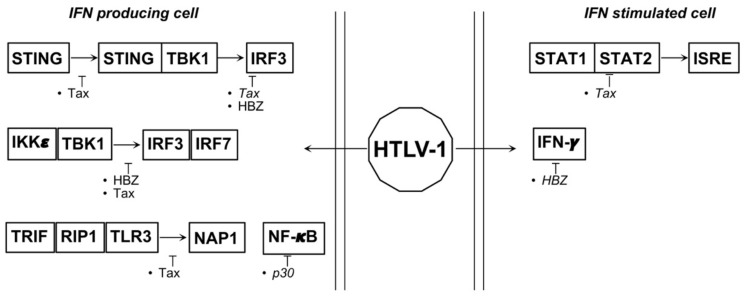
Proteins and miRNAs able to deregulate type I IFNs relevant pathways during HTLV-1 associated tumorigenesis. Italics represent indirect action.

Based on the ability of these viruses, particularly HTLV-1, to induce an inflammatory disease as HAM/TSP, it was not surprising to reveal that HTLV-1 interferes with the IFNs system. Tax and HBZ proteins are not only the HTLV-1 oncogenes responsible for transformation and the maintenance of the transformed phenotype, but they are also the “tools” that the virus uses to block upstream and downstream IFN signaling. Indeed, it has been reported that Tax inhibits the hyper-phosphorylation of IRF3 through the interaction with TBK-1 and IKKε [72]. Tax is also able to interact with STING, thereby blocking the cGAS-STING axis leading to IRF3 activation and IFNβ gene induction [72]. A similar inhibitory activity of Tax was reported also for TRIF and receptor interacting protein 1 (RIP-1) kinase thereby impeding IRF7 activation and, by consequence, transcriptional activation of type I IFNs genes [73]. Through the activation of the NF-κB signaling pathway, Tax induces the expression of SOCS-1 but, contrary to what one might think, it uses this inhibitory protein to block the innate antiviral signaling rather than the IFN signal transduction [74]. Indeed, SOCS-1 siRNA experiments performed on 293-T cells transfected with reporter constructs together with Tax, showed that SOCS-1 silencing is unable to block the Tax-dependent inhibition of IFNα signaling, whereas it was able to block the Tax-dependent inhibition of an IFNβ reported gene stimulated by PolyI:C [74]. It was demonstrated that SOCS1 inhibited IFN-β production by targeting IRF3 for ubiquitination and proteasomal degradation indicating that Tax uses this pathway to inhibit IFNβ synthesis [75]. Tax is also able to block the IFN signaling through a competitive usage of CBP/p300 with STAT2. Nonetheless, Tax does not affect the formation of the ISGF3 complex on the ISRE containing promoters [76] (Figure 3).

HBZ plays a dual role in the IFNs system. If, on the one hand, as reported for Tax, HBZ is able to inhibit the activation of IRF3, on the other hand, this viral factor enhances the transcriptional activity of IRF7 [77]. Overexpression and co-immunoprecipitation experiments demonstrated that HBZ physically associates with a complex containing both IKKε and IRF7 suggesting a role in the phosphorylation of this transcription factor, whereas HBZ is neither able to bind TBK-1 or IRF3, nor to inhibit IRF-3 hyperphosphorylation, suggesting that HBZ blocks IRF3 transactivating activity [77]. Interestingly enough, HBZ is also effective on Type II IFNs, as demonstrated using HBZ Tg mice [78]. Indeed, in this experimental model, it was reported that the challenge with two different Th1 pathogen inducers (i.e., L. Monocytogenes and Herpes Simplex Virus type 2) was efficiently restrained at the beginning of the infection but not at late steps. This suggests that the innate immune response is effective in Tg mice, but the cell-mediated acquired immunity is impaired. Cytokine measurement demonstrated lower levels of Th1 cytokines as TNFα, IL-2 and, especially, IFNγ but not, IL-6 and IL-10. At the molecular level, the authors demonstrated that HBZ impairs both NF-ATc and AP-1 binding to the IFNγ promoter, thereby interfering with their transcriptional activity [78].

Besides T cells, HTLV-1 infects monocytes/macrophages and dendritic cells (DCs) as well [79,80], leading to impaired antigen-presenting functions and IFNα release [81]. It was reported that in myeloid cells HTLV-1 impairs type I IFNs production mainly through p30. This accessory viral protein is located at the nuclear level and is able to interact and block the myeloid-specific transcriptional factor PU.1 [82], leading to a profound inhibition of TLRs downstream signaling (i.e., TLR3 and -4) which results not only in the downregulation of TLR4, TNFα, MCP-1, IL-8, and IL-12 expression, but also in reduced transcriptional activation of IFNα1 and IFNβ promoters and, by consequence, reduced type I IFN release and expression of MxA, OAS and APOBEC3G [83] (Figure 3).

HTLV-1 interferes with the IFN system also through the modulation of miRNAs in target cells. Indeed, miR-155 is upregulated in HTLV-1^+^ patients [84] and is able to favor the upregulation of IFNγ in natural killer cells [30].

### 4.3. MCPyV and JCPyV

Four members of the Polyomaviridae family Merkel cell polyomavirus (MCPyV) BK virus (BKPyV), John Cunningham virus (JCPyV), and trichodysplasia spinulosa polyomavirus (TSPyV) have been associated with the development of specific malignant tumors [85]. Molecular and epidemiological evidence only supports the role of MCPyV as a carcinogen to humans. Despite the ability of these viruses to induce different pathologies, (i.e., MCPyV induces Merkel Cell Carcinoma, BKV a Polyomavirus Nephropathy and JCV a Progressive Multifocal Leukoencephalopathy) all these viruses share the characteristic to exert their pathogenic activity only in immunocompromised hosts [86,87,88]. The oncogenic mechanisms of MCPyV are already a matter of investigation. No direct oncogenic effect of viral proteins was reported, nor MCPyV has been reported to exploit cellular oncogenic mechanisms (p53, PTEN, Raf, Ras, etc.) [89]. Based on data showing an increase of MCC upon UV irradiation, a possible involvment of the DNA repair mechanism was hypothesized [90].

Very few data are available in literature about the ability of these viruses to interplay with the IFN system and this is especially true for MCPyV. Regarding non-oncogenic Polyomaviruses some reports indicate that in vitro cell infection leads to ISGs induction and antiviral state establishment. This is the case of JCV that, following expression in primary human fetal glial (PHFG) cells, induces a set of 51 genes, among which most of them (15 out of 51) are ISGs [91]. Further, the treatment of JCV-expressing PHFG cells with IFNα or IFNβ restricts JCV replication [92]. Similar results were also obtained for IFNγ [93]. Even BKV is efficiently suppressed by IFNγ [94]. Based on these observations, it is not surprising to find that MCPyV replication is also effectively inhibited by both type I and II IFNs, mainly through downmodulation of large T antigen (LTA) expression and apoptosis induction [95].

**Figure 4:**
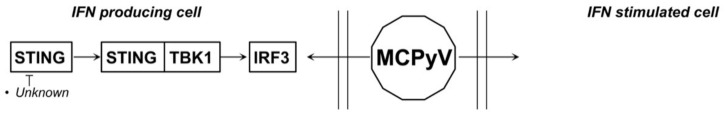
Proteins and miRNAs able to deregulate type I IFNs relevant pathways during MCPyV associated tumorigenesis. Italics represent indirect action.

In 2020 it was reported a possible mechanism of action of MCPyV antiviral escape based on STING downmodulation [96]. Indeed, if compared to MCPyV negative cells, MCPyV positive carcinoma cell lines express very low levels of STING, and reintroduction of an agonist-inducible form of STING into these cells sensitizes them to cell death. Nonetheless, the molecular details allowing STING downmodulation in MCPyV positive carcinoma cells are still elusive. Collectively, these observations suggest that this virus (as well as his relatives) is unable to counteract the antiviral response generated by IFNs unless in the case of immunocompromised hosts (Figure 4).

### 4.4. Oncogenic Herpesviruses

Herpesviridae represent a large family of structurally and genetically correlated DNA viruses belonging to three main genera: α-, β- and γ-herpesviruses. Between these, only the members of γ-herpesviruses genus as Kaposi’s sarcoma-associated Herpesvirus (KSHV) and Epstein–Barr Virus (EBV) retain an oncogenic potential. Indeed, KSHV is associated with endothelial Kaposi’s sarcoma and primary effusion lymphoma (PEL), whereas EBV can cause Burkitt’s and Hodgkin’s lymphomas as well as nasopharyngeal carcinoma [97,98]. All the members of this virus family express a plethora of products able to deeply reprogram the host cells thereby allowing the virus to begin, depending on the context, a progeny productive lytic cycle or a latent cellular infection [99]. The different latency program induced is tightly connected to the transformation process due to the different set of viral products expressed. Using lymphoblastoid cell lines (LCLs) as a surrogate in vitro model of EBV-induced B-cell transformation, it has been demonstrated that (Epstein–Barr nuclear antigen) EBNA2, EBNALP, EBNA3A, EBNA3C, and latent membrane protein 1 (LMP1) play an essential role for efficient B-cell transformation by mimicking CD40 (LMP1) and BCR signaling (LMP2A), facilitating p53 degradation (EBNA1), blocking apoptosis trough different mechanisms (EBNA3C, LMP1, and LMP2A) and inducing a deep reprogramming of cell gene expression (EBNA2A in association with EBNALP and EBNA2) [19]. The distinct type of latency also has an impact on the type I IFN system due to the fact that many of the proteins driving the different latent programs are involved in the regulation of the antiviral response.

Many of the key proteins involved in the pathogen sensing and in the induction of type I IFNs are targeted by Herpesviruses viral products. Indeed, it has been reported that the EBV deconjugase BPLF1, interacts with TRIM25 and members of the 14-3-3 family, thereby promoting TRIM25 autoubiquination, inhibition of RIG-I signalosome, and type I IFNs response [100,101,102]. This feature is shared also with KSHV-ORF64 and the UL-48 protein expressed by the non-oncogenic Herpesvirus HCMV [102]. RIG-I is targeted also by LMP1 through a proteasome-dependent, MG32-sensitive pathway [103]. Not only the TLRs/TRAFs/TRIM axis, leading to RIG-I activation, is affected by Herpesviruses but also the MAVS/STING/GAS axis is targeted. In particular, EBV protein BHRF1, a BCL2 homolog, triggers autophagy and mitophagy, leading to the prevention of MAVS and STING activation and, as a consequence, IFNβ induction [104]. Independently of the PRRs involved, all the signaling pathways converge to IKKε/TBK1 complex thereby inducing IRF3 hyperphosphorylation, dimerization, and nuclear translocation. Herpesviruses interfere also with this step. In this case, EBV protein BGLF4 interacts with IRF3 and prevents IFNβ promoter transactivation without inhibiting IRF3 phosphorylation and nuclear translocation [105]. Not only IRF3 but also IRF7, able to interact with the former to induce the transcriptional activity of IFNα genes, is targeted by several Herpesvirus products even if, in this case, positive and negative regulations occur. Indeed, while LMP1 activates IRF7 and IFNα/β production [106], BamHI Z fragment leftward open reading frame 1 (BZLF1) counteracts this action [107]. Since BZLF1 is expressed during lytic cycle whereas LMP1 is expressed during latency, it was hypothesized that type I IFNs induction mediated by LMP1 is functional to maintain latency whereas, when lytic cycle is induced, BZLF1 allows IFNs production inhibition. Further, IRF7 is also targeted by LF2, another EBV tegument protein, leading to suppression of IFNα production and IFN-mediated immunity [108].

Once synthesized and released, IFNs interact with their cognate receptors thereby activating JAK-STATs pathway which is, again, targeted by Herpesviruses. LMP-1 interacts with and negatively affects Tyk2 phosphorylation and IFN signaling in human B cells [109]. Tyk2 is also targeted by BGLF2 probably through its C-terminal domain [110]. Both LMP2A and 2B of EBV act upstream of Tyk2, by targeting interferon receptors for degradation. Although LMP2A and LMP2B do not affect the IFNRs cell-surface expression, they accelerated their turnover through a process involving endosome degradation [111].

Finally, EBV is able not only to counteract activating signals but induces also negative regulators of the IFNs signaling. This is the case of SOCS3 which is induced by BZLF1/ZEBRA protein, leading to Tyk2/JAK1 inactivation in monocytes without interfering with type I IFNs production and release [112].

**Figure 5:**
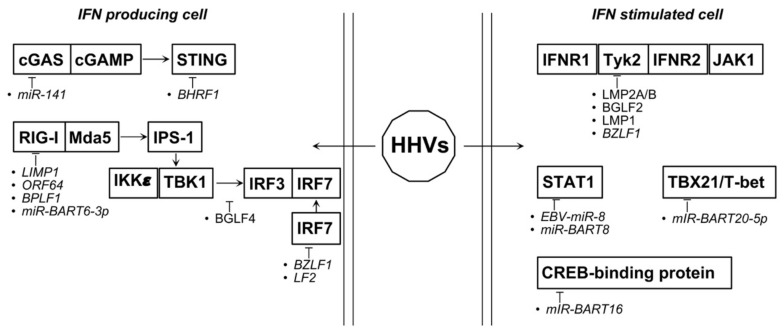
Proteins and miRNAs able to deregulate type I IFNs relevant pathways during HHV-associated tumorigenesis. Italics represent indirect action.

EBV exploits B cell receptor (BCR) for lytic reactivation in B cells. It is well known that FOXO3 protein dampens type I IFNs response through the suppression of IRF7. EBV regulates the FOXO3 activities through the expression of miR-BART9 and the upregulation on human miR-141 thus targeting ZCCHC3, a cosensor for cGAS protein to manage the shift from latent to lytic life cycle [113] (Figure 5).

During chronic EBV infection, several miRNAs are upregulated. Among these miR-494-3P and miR-142-3P lead to AKT activation and suppression, respectively. EBV life cycle is strictly linked to the human antiviral mechanisms, particularly through exploitation of its microRNA fine-tuning. For example, BART20-5P inhibits IFN-γ while EBV-miR-8 inhibits STAT1 [114].

During latent infection, EBV activities are almost repressed, but EBV miRNAs are highly expressed. Lu et al. focalize their attention on miR-BART6-3p that targets the 3′UTR of RIG-I mRNA leading to the deactivation of type I IFNs response. Similarly, miR-BART20-5p targets TBX21/T-bet to downmodulate IFNγ production. Redundance and opposition in miRNA activities is typical in EBV, in fact among the highly expressed EBV non-coding RNAs synthesized during the latent period, EBERs induce type I IFN response [115].

To favor the latent period persistence, EBV highly produces miR-BART16. In EBV-transformed B cells and gastric carcinoma cells, such viral miRNA disables cells to respond to the IFNα stimulation toward the targeting a key transcriptional coactivator of IFN signaling named CREB-binding protein [116].

Additional viral miRNAs that favor a prolonged viral infection are miR-BART8 and miR-BART20-5p. Both miRNAs deregulate IFN-γ/STAT1 pathway, but miR-BART8 inhibits only STAT1 whereas miR-BART20-5p can target T-bet and IFN-γ with secondary suppression of STAT1. The deregulation of STAT1 leads to TP53 and miR-let7a suppression, thereby inducing progression of nasal NK-cell lymphoma (NNL) [117] (Figure 5).

Very little is known about the role of extracellular vesicles during HHV-associated carcinogenesis. A work by Meckes Jr. et al. shows the proteomic characterization of exosome protein cargo derived from EBV or KSHV positive human cells. An overlook about the exosome content demonstrates that EBV and KSHV exosomes have multiple common molecular mediators (viral miRNAs and viral proteins, in particular). By hypothesizing that exosomes could transfer biologically active signal transduction components into host uninfected cells, the researchers establish that KSHV exosomes could preferentially modulate cellular metabolism while EBV exosomes could deregulate several pathways, namely TP53, JAK/STAT, NF-κB, IRF7, and MAPK through the presence of LIMP1 [118].

### 4.5. HBV and HCV

Hepatocellular carcinoma (HCC) is the most common primary liver cancer, the sixth most common cancer, and the second leading cause of cancer deaths worldwide. In Asia, chronic hepatitis B virus (HBV, Hepadnaviridae family, Orthohepadnavirus genus) infection is the primary cause of HCC, while in the Western world, besides alcoholic cirrhosis and non-alcoholic steatohepatitis (NASH) chronic hepatitis C virus (HCV, Flaviviridae family, Hepacivirus genus) is the main cause [119]. Chronic inflammation plays a major role in HCC initiation and progression, but hepatitis viruses can also directly drive liver cancer. HBV DNA integration into host cell genome, metabolic reprogramming, induction of the cellular stress response pathway and interference with tumor suppressor genes are the main mechanisms used by hepatitis viruses to induce HCC. HBV mainly uses the X protein (HBx) not only as a viral transcriptional trans-activator but also to cis- and trans-activate a group of so-called HCC-associated genes. On the other hand, HCV induces cellular transformation through several mechanisms among which p53 interference by NS3 and Core protein, pRb sequestration by NS5B, and trans-activation of HCC-associated genes by Core protein are included (for a detailed review see [120]).

Despite their differences in terms of structure, genome, and viral replication cycle, both HBV and HCV have evolved strategies to neutralize the IFN-based antiviral response as attested by studies in animal models and observations in patients [121,122,123,124]. If on the one hand, this phenomenon is important from the pathogenetic/mechanistic point of view, on the other hand, it is also a major concern from the therapeutic point of view because in anti-HBV therapeutic schedule, standard or pegylated IFNs is used alone and in combination with nucleotide analogs and therapeutic failure is associated to this feature [125].

STAT1 represents the main target for both hepatic viruses. It has been reported that HBV and HCV do not interfere with tyrosine phosphorylation nor with nuclear translocation of STAT1 but with its ability to bind IFN responsive elements, thereby inducing the transactivation of ISG promoters [122,123]. This activity is mediated by the protein phosphatase 2A (PP2A) that acts upstream of the protein arginine N-methyltransferase 1 (PMRT1). PMRT1 activation allows the arginine methylation of STAT1, its association with E3 SUMO-protein ligase PIAS1 and the disruption of DNA binding proprieties of STAT1 [123,126]. In vitro data were corroborated by in vivo observation on hepatic biopsies demonstrating elevated expression of PP2A and increased STAT1 methylation on these specimens. In the case of HBV, it has been demonstrated that PP2A upregulation is mediated by both X and S proteins [123]. HBV blocks Jak-STAT signaling through the induction, mediated by HBx, of SOCS3 [127].

Besides these similarities, HBV is also able to prevent the strong immune response associated with type I IFN production [128]. The interaction between the viral particle and the target cells rapidly leads to innate immune response inhibition [129], leading to the concept of HBV as a “stealth” virus. Hbs- and HbeAg are both able to suppress type I IFN production mediated by MyD88-dependent pathways by interfering with the interaction between MyD88 and the major vault protein (MVP), a virus-induced cellular protein that plays a fundamental role in the IFN production [130,131]. HBV Pol has also a signaling “inhibitory” function on STING as it leads to a decreased Lys63 polyubiquitination through direct interaction and, by consequence, to a decrease in IRF3 activation and IFNβ production. Pol is also responsible for the innate response evasion through the interaction with the DEAD box RNA helicase DDX3 and the inhibition of TBK1/IKKε activity [132]. Even HbcAg is involved in the downregulation of IFNβ production probably through interference with TBK1 activation [133].

**Figure 6:**
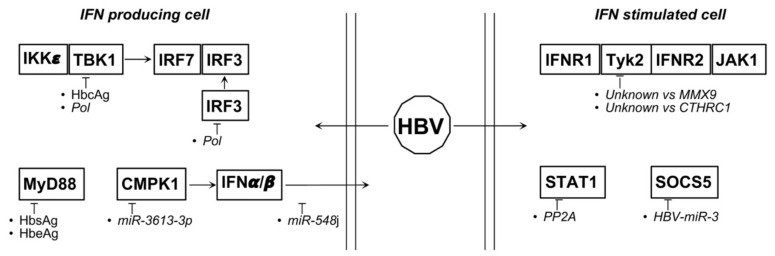
Proteins and miRNAs able to deregulate type I IFNs relevant pathways during HBV associated tumorigenesis. Italics represent indirect action.

HBV interferes with IFN signaling even upstream of STAT1 activation. It has been reported that in cancer tissue an elevated expression of collagen triple helix repeat containing 1 (CTHRC1) leads to IFNAR1/2 reduced expression and JAK/STAT signaling repression [134]. Similarly, in macrophages it has been demonstrated that HBV induces the expression of matrix metalloprotease 9 (MMX9) which, in turn, binds to IFNAR1, leading to its downmodulation [135] (Figure 6).

Whereas HBV blocks pathways of both IFN-I production and activity, HCV inhibits mainly its production. Besides STAT1 inhibition it has been reported that core protein is able to inhibit the expression of several ISGs such as IRF1, IL-15, IL-12, and PKR [136]. PKR antiviral activity is targeted also by E2 envelope protein that is able to prevent the PKR-dependent phosphorylation of eIF2α, thereby inhibiting protein synthesis repression induced by PKR [137]. Additionally, NS5A targets PKR by direct binding blocking both autophosphorylation and eIF2α phosphorylation [138] and, in addition, binds to 2-5OAS thereby preventing the activation of the 2-5OAS/RNAse L system [139].

**Figure 7:**
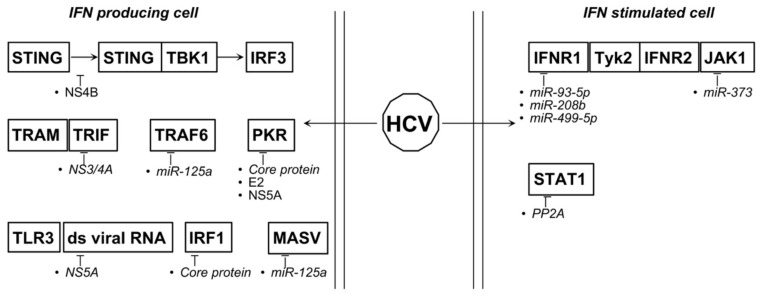
Proteins and miRNAs able to deregulate type I IFNs relevant pathways during HCV associated tumorigenesis. Italics represent indirect action.

The only inhibitory function of HCV viral products able to interfere with the IFNβ synthesis is attributable to NS3/4A and NS4B. The former is the viral protease that facilitates the cleavage of both MASV and TRIF, key players in the induction of IFNβ [140,141]. On the other side, NS4B sequesters STING, thus impeding STING/TBK1 association and, by consequence, IFNβ induction [142,143] (Figure 7).

Both HBV and HCV interfere with the IFN system also through the modulation of miRNAs. In particular, it has been reported that in chronic hepatitis B (CHB) patients monocytes-derived dendritic cells overexpress miR-548 family members, especially miR-548j, and this is correlated to a reduced IFNα/β release [144]. MiR-3613-3p is also upregulated by HBV, thereby inducing the downregulation of cytidine/uridine monophosphate kinase 1 (CMPK1) and, again as a consequence, IFNα/β release [145]. HBV inhibits miR-1287-5p through the induction of circular RNA circ_0004812. This circRNA sponges miR-1287-5p and enhances the expression of follistatin-related protein 1 (FSRT1) thus impairing IFN-induced immune response [146]. Finally, HBV expresses its own miRNAs, as HBV-miR-3, and it has been suggested that HBV-miR-3 plays a role in the establishment of chronic infection and latency through a mechanism involving SOCS5 downmodulation and IFNα/β release in hepatocytes. HBV-miR-3 is also released into exosomes and induces M1 polarization in monocyte/macrophage, again through the inhibition of SOCS5-mediated ubiquitination of EGFR [147] (Figure 6).

Regarding HCV, several groups reported both up- and downmodulation of different miRNAs. Among upregulated miRNAs there are mir-93-5p, -125a, -208, -373 and -499a-5p. HCV core protein upregulates miR-93-5p expression, leading to both IFNAR1 downmodulation and STAT1 ipo-phosphorylation [148]. IFNAR1, together with IFNλ II and III, is targeted also by miR-208b and miR-499a-5p [149]. Similarly, mir-373 blocks type I IFNs signaling by targeting JAK1, IRF5, and -9 and, again, STAT1 phosphorylation [150,151]. Mir-125a acts, instead, upstream of IFN synthesis, being able to downmodulate both MASV and TRAF6 [152]. MiR181a-2-3p, -374a-3p, -374a-5p, -204-5p and -146b-5p are downmodulated in peripheral blood mononuclear cells (PBMCs) isolated from HCV positive chronic patients [153]. In particular, it has been demonstrated that the expression of HCV core protein leads to miR-146b-5p downregulation in monocytes/macrophages and that this modulation is responsible for the mRNA induction of IL-10, TGFβ, and CXCL10, the mRNA downregulation of IFNα, IL-12, and TNFα, as well as the activation of the NF-κB signaling [153] (Figure 7).

**Table 1:** MicroRNAs linked to IFNs response in virus-induced tumorigenesis.

Oncoviruses	MicroRNAs ^1^	Targets/Function ^2^	Refs.
**HPV**	miR-122 ↑	E6 ↓	[64]
	miR-129-5P ↑	HPV18 E6 and E7 ↓	[63]
	miR-221 ↑	SOCS ↓	[65]
	miR-10a, -18a, -19a, -21, -34a, -98, -182, -194, -590-5p	Exploitation of some E6 and E7 activities	[62]
	miR-34a ↑	p53 ↓	[37]
**HTLV-1**	miR-155 ↑	IFNγ upregulation	[30]
**EBV**	miR-BART92	FOXO3 ↓	[113]
	miR-141 ↑	ZCCHC3 ↓	[113]
	miR-494-3P ↑	AKT activation	[114]
	miR-142-3P ↑	AKT suppression	[114]
	miR-BART20-5P2	TBX21/T-bet ↓	[115]
	EBV-miR-82	STAT1 ↓	[114]
	miR-BART6-3p2	RIG-I ↓	[115]
	EBERs2	induce IFN I response	[115]
	miR-BART162	CREB-binding protein ↓	[116]
	miR-BART82	STAT1 ↓	[117]
	miR-let7a ↓	progression of NNL	[117]
**HBV**	miR-548j ↑	less IFNα/β release	[144]
	miR-3613-3p ↑	CMPK1 ↓	[145]
	miR1287-5p ↓	FSRT1 ↑	[146]
	HBV-miR-32	SOCS-5 ↓	[147]
**HCV**	miR-93-5p ↑	IFNAR1 ↓	[148]
	miR-208b ↑	IFNAR1, IFNλ ↓	[149]
	miR-499a-5p ↑	IFNAR1, IFNλ ↓	[149]
	mir-373 ↑	JAK1, IRF5 and -9 ↓	[150,151]
	mir-125a ↑	MASV, TRAF6 ↓	[152]
	miR181a-2-3p, -374a-3p, -374a-5p, -204-5p ↓		[153]
	miR-146b-5p ↓	activation NF-κB signaling	[153]

^1^ Listed miRNAs refer to viral induced (↑) or repressed (↓) cellular miRNAs. miR-BART20-5P, EBV-miR-8, miR-BART6-3p, EBERs, miR-BART16 and miR-BART8 are EBV-expressed miRNAs, whereas HBV-miR-3 is expressed by HBV. Reported data refer to papers published from 2015 up to now. For data reported before 2015 see also [3]; ^2^ Listed Target/Functions point to the affected protein/function by the corresponding miRNA. ↑ indicates the induction of Protein/Function, whereas ↓ the down-modulation.

## 5. Concluding Remarks

Significant evidence shows that the innate response to virus infection and in particular IFN-mediated biology are important regulators of virus-induced tumorigenesis. Nevertheless, further studies are needed to determine the exact mechanisms of cancer induced by oncogenic viruses. The studies reviewed here provide new insights into regulatory mechanisms between virus infection, tumorigenesis, and the IFN system in host cells (Figure 1).

In recent years, there has been accumulating evidence about the complex regulation of the IFN system, showing that non-STAT pathways play important and essential roles in IFN-signaling. This has led to an evolution of our understanding of the complexity associated with IFN activity and how interacting signaling networks determine the relevant IFN response. Not surprisingly, miRNAs have been identified that target IFNs and ISGs. Cellular miRNAs together with virally encoded miRNAs have been reported to interfere with IFN or ISGs to regulate their expression and thus limit their function, (Table 1). In any case, the picture, still lacking, that emerges from all the results regarding the miRNAs dysregulation in cancers shows a complexity of pathways involved in immune response and tumor progression. Certainly, the contribution of miRNAs to an IFN response is important, cell-dependent, and involves a network of interactions, affecting both the production of IFNs and the activity of the signaling responses.

In the context of Human papillomavirus infection, we and other authors reported that EVs released by HPV expressing cells participate in intercellular communication to transfer microRNAs between virus-infected cells and neighboring normal cells possibly modifying the microenvironment, affecting tumor development and immune resistance. Tumorigenesis, as a final outcome of oncogenic virus infection, is therefore dependent on a fine balance of pro- and anti-viral factors, comprising IFNs, ISGs, cellular and viral miRNAs machinery, as well as cellular communication and microenvironment modification via classical secretion mechanisms and EV-mediated delivery.

Finally, type I IFNs clearly appear as central coordinators of innate immune responses targeted during tumorigenesis by the different oncoviruses described. It seems less clear how IFNs may trigger immune suppressive mechanisms in cancer-promoting malignant progression and resistance to therapies. More studies need to be performed to further elucidate how an IFN system is integral to this complex picture and possibly might be a useful therapeutic target.

## Data Availability

Not applicable.

## Abbreviations

Activating Transcription Factor 3 (ATF3); Apolipoprotein B mRNA editing enzyme, catalytic polypeptide-like 3G (APOBEC3G); CCAAT-Enhancer-Binding Protein α (C/EBPα); cyclic GMP–AMP synthase (cGAS)–Stimulator of Interferon Genes (STING)**;** Eukaryotic Initiation Factor 2 subunit α (eIF2α); Hepatitis B Virus (HBV); Hepatitis C Virus (HCV); Human Papillomavirus (HPV); Human T-Lymphotropic Virus type 1 (HTLV-1); Interferon-induced Protein 44-like (IFI44L); IFN-Stimulated Growth Factor 3 (ISGF3); IFN-Stimulated Response Element (ISRE); IFN Regulatory Factor (IRF); Inhibitor of nuclear factor κB Kinase-ε (IKKε); Janus kinase 1 (JAK1); John Cunningham virus (JCPyV); Laboratory of Genetics and Physiology-2 (LGP2); Mechanistic Target of Rapamycin (mTOR); Melanoma Differentiation-Associated gene 5 (MDA5); Merkel Cell Polyomavirus (MCPyV); Mitochondrial AntiViral Signaling protein (MAVS); Mitogen-Activated Protein Kinase (MAPK); Myeloid Differentiation primary response 88 (MyD88); Myxovirus resistance 1 (MX1); NOD-Like Receptor member X1 (NLRX1); 2′-5′-Oligoadenylate Synthetase 1 (OAS1); Pattern Recognition Receptors (PRRs); Protein Kinase C (PKC); Protein Kinase Receptor (PKR); Retinoic acid-Inducible Gene 1 (RIG-I); Signal Transducer and Activator of Transcription (STATs); Suppressors of Cytokine Signaling (SOCS); Toll-Like Receptors (TLRs); TIR-domain-containing adapter-inducing interferon-β (TRIF); Tripartite Motif Family Protein (TRIM); Tumor Necrosis Factor Receptor-Associated Factor 3 (TRAF3); TRAF family member-associated NF-κB activator (TANK)-binding kinase 1 (TBK1); Transcription Nuclear Factor-κB (NF-κB); Tyrosine kinase 2 (Tyk2).

## References

[1] Bekisz J., Baron S., Balinsky C., Morrow A., Zoon K.C. (2010). Antiproliferative Properties of Type I and Type II Interferon. Pharmaceuticals.

[2] Chiantore M.V., Vannucchi S., Mangino G., Percario Z.A., Affabris E., Fiorucci G., Romeo G. (2009). Senescence and cell death pathways and their role in cancer therapeutic outcome. Curr. Med. Chem..

[3] Fiorucci G., Chiantore M.V., Mangino G., Romeo G. (2015). MicroRNAs in virus-induced tumorigenesis and IFN system. Cytokine Growth Factor Rev..

[4] Fiorucci G., Chiantore M.V., Mangino G., Percario Z.A., Affabris E., Romeo G. (2012). Cancer regulator microRNA: Potential relevance in diagnosis, prognosis and treatment of cancer. Curr. Med. Chem..

[5] Raab-Traub N., Dittmer D.P. (2017). Viral effects on the content and function of extracellular vesicles. Nat. Rev. Microbiol..

[6] Mesev E.V., LeDesma R.A., Ploss A. (2019). Decoding type I and III interferon signalling during viral infection. Nat. Microbiol..

[7] Crosse K.M., Monson E.A., Beard M.R., Helbig K.J. (2018). Interferon-Stimulated Genes as Enhancers of Antiviral Innate Immune Signaling. J. Innate Immun..

[8] Mazewski C., Perez R.E., Fish E.N., Platanias L.C. (2020). Type I Interferon (IFN)-Regulated Activation of Canonical and Non-Canonical Signaling Pathways. Front. Immunol..

[9] Crouse J., Kalinke U., Oxenius A. (2015). Regulation of antiviral T cell responses by type I interferons. Nat. Rev. Immunol..

[10] Makris S., Paulsen M., Johansson C. (2017). Type I Interferons as Regulators of Lung Inflammation. Front. Immunol..

[11] Dutta S., Das N., Mukherjee P. (2020). Picking up a Fight: Fine Tuning Mitochondrial Innate Immune Defenses Against RNA Viruses. Front. Microbiol..

[12] Duic I., Tadakuma H., Harada Y., Yamaue R., Deguchi K., Suzuki Y., Yoshimura S.H., Kato H., Takeyasu K., Fujita T. (2020). Viral RNA recognition by LGP2 and MDA5, and activation of signaling through step-by-step conformational changes. Nucleic Acids Res..

[13] Lee S., Channappanavar R., Kanneganti T.D. (2020). Coronaviruses: Innate Immunity, Inflammasome Activation, Inflammatory Cell Death, and Cytokines. Trends Immunol..

[14] Louis C., Burns C., Wicks I. (2018). TANK-Binding Kinase 1-Dependent Responses in Health and Autoimmunity. Front. Immunol..

[15] Oshiumi H., Kouwaki T., Seya T. (2016). Accessory Factors of Cytoplasmic Viral RNA Sensors Required for Antiviral Innate Immune Response. Front. Immunol..

[16] Zanin N., Viaris de Lesegno C., Lamaze C., Blouin C.M. (2020). Interferon Receptor Trafficking and Signaling: Journey to the Cross Roads. Front. Immunol..

[17] Stark G.R., Cheon H., Wang Y. (2018). Responses to Cytokines and Interferons that Depend upon JAKs and STATs. Cold Spring Harb. Perspect. Biol..

[18] Raftery N., Stevenson N.J. (2017). Advances in anti-viral immune defence: Revealing the importance of the IFN JAK/STAT pathway. Cell. Mol. Life Sci..

[19] Saha A., Robertson E.S. (2019). Mechanisms of B-Cell Oncogenesis Induced by Epstein-Barr Virus. J. Virol..

[20] Umbach J.L., Cullen B.R. (2009). The role of RNAi and microRNAs in animal virus replication and antiviral immunity. Genes Dev..

[21] Lever A.M., Jeang K.T. (2011). Insights into cellular factors that regulate HIV-1 replication in human cells. Biochemistry.

[22] Houzet L., Jeang K.T. (2011). MicroRNAs and human retroviruses. Biochim. Biophys. Acta.

[23] Platanias L.C. (2005). Mechanisms of type-I- and type-II-interferon-mediated signalling. Nat. Rev. Immunol..

[24] Kohanbash G., Okada H. (2012). MicroRNAs and STAT interplay. Semin. Cancer Biol..

[25] Li A., Song W., Qian J., Li Y., He J., Zhang Q., Li W., Zhai A., Kao W., Hu Y. (2013). MiR-122 modulates type I interferon expression through blocking suppressor of cytokine signaling 1. Int. J. Biochem. Cell Biol..

[26] Su C., Hou Z., Zhang C., Tian Z., Zhang J. (2011). Ectopic expression of microRNA-155 enhances innate antiviral immunity against HBV infection in human hepatoma cells. Virol. J..

[27] Collins A.S., McCoy C.E., Lloyd A.T., O’Farrelly C., Stevenson N.J. (2013). miR-19a: An effective regulator of SOCS3 and enhancer of JAK-STAT signalling. PLoS ONE.

[28] Wiesen J.L., Tomasi T.B. (2009). Dicer is regulated by cellular stresses and interferons. Mol. Immunol..

[29] Ostermann E., Tuddenham L., Macquin C., Alsaleh G., Schreiber-Becker J., Tanguy M., Bahram S., Pfeffer S., Georgel P. (2012). Deregulation of type I IFN-dependent genes correlates with increased susceptibility to cytomegalovirus acute infection of dicer mutant mice. PLoS ONE.

[30] Trotta R., Chen L., Ciarlariello D., Josyula S., Mao C., Costinean S., Yu L., Butchar J.P., Tridandapani S., Croce C.M. (2012). miR-155 regulates IFN-γ production in natural killer cells. Blood.

[31] Noguchi S., Yamada N., Kumazaki M., Yasui Y., Iwasaki J., Naito S., Akao Y. (2013). socs7, a target gene of microRNA-145, regulates interferon-β induction through STAT3 nuclear translocation in bladder cancer cells. Cell Death Dis..

[32] Polioudakis D., Bhinge A.A., Killion P.J., Lee B.K., Abell N.S., Iyer V.R. (2013). A Myc-microRNA network promotes exit from quiescence by suppressing the interferon response and cell-cycle arrest genes. Nucleic Acids Res..

[33] Buggele W.A., Horvath C.M. (2013). MicroRNA profiling of Sendai virus-infected A549 cells identifies miR-203 as an interferon-inducible regulator of IFIT1/ISG56. J. Virol..

[34] Li Y., Xie J., Xu X., Wang J., Ao F., Wan Y., Zhu Y. (2013). MicroRNA-548 down-regulates host antiviral response via direct targeting of IFN-λ1. Protein Cell.

[35] Honegger A., Schilling D., Bastian S., Sponagel J., Kuryshev V., Sültmann H., Scheffner M., Hoppe-Seyler K., Hoppe-Seyler F. (2015). Dependence of intracellular and exosomal microRNAs on viral E6/E7 oncogene expression in HPV-positive tumor cells. PLoS Pathog..

[36] Chiantore M.V., Mangino G., Iuliano M., Zangrillo M.S., De Lillis I., Vaccari G., Accardi R., Tommasino M., Columba Cabezas S., Federico M. (2016). Human papillomavirus E6 and E7 oncoproteins affect the expression of cancer-related microRNAs: Additional evidence in HPV-induced tumorigenesis. J. Cancer Res. Clin. Oncol..

[37] Iuliano M., Mangino G., Chiantore M.V., Zangrillo M.S., Accardi R., Tommasino M., Fiorucci G., Romeo G. (2018). Human Papillomavirus E6 and E7 oncoproteins affect the cell microenvironment by classical secretion and extracellular vesicles delivery of inflammatory mediators. Cytokine.

[38] Tommasino M. (2014). The human papillomavirus family and its role in carcinogenesis. Semin. Cancer Biol..

[39] Pal A., Kundu R. (2019). Human Papillomavirus E6 and E7: The Cervical Cancer Hallmarks and Targets for Therapy. Front. Microbiol..

[40] Westrich J.A., Warren C.J., Pyeon D. (2017). Evasion of host immune defenses by human papillomavirus. Virus Res..

[41] Scott M., Nakagawa M., Moscicki A.B. (2001). Cell-mediated immune response to human papillomavirus infection. Clin. Diagn. Lab. Immunol..

[42] Karim R., Meyers C., Backendorf C., Ludigs K., Offringa R., van Ommen G.J., Melief C.J., van der Burg S.H., Boer J.M. (2011). Human papillomavirus deregulates the response of a cellular network comprising of chemotactic and proinflammatory genes. PLoS ONE.

[43] Chang Y.E., Laimins L.A. (2000). Microarray analysis identifies interferon-inducible genes and Stat-1 as major transcriptional targets of human papillomavirus type 31. J. Virol..

[44] Nees M., Geoghegan J.M., Hyman T., Frank S., Miller L., Woodworth C.D. (2001). Papillomavirus type 16 oncogenes downregulate expression of interferon-responsive genes and upregulate proliferation-associated and NF-kappaB-responsive genes in cervical keratinocytes. J. Virol..

[45] Li T., Chen Z.J. (2018). The cGAS-cGAMP-STING pathway connects DNA damage to inflammation, senescence, and cancer. J. Exp. Med..

[46] Motwani M., Pesiridis S., Fitzgerald K.A. (2019). DNA sensing by the cGAS-STING pathway in health and disease. Nat. Rev. Genet..

[47] Lau L., Gray E.E., Brunette R.L., Stetson D.B. (2015). DNA tumor virus oncogenes antagonize the cGAS-STING DNA-sensing pathway. Science.

[48] Ronco L.V., Karpova A.Y., Vidal M., Howley P.M. (1998). Human papillomavirus 16 E6 oncoprotein binds to interferon regulatory factor-3 and inhibits its transcriptional activity. Genes Dev..

[49] Shaikh M.H., Bortnik V., McMillan N.A., Idris A. (2019). cGAS-STING responses are dampened in high-risk HPV type 16 positive head and neck squamous cell carcinoma cells. Microb. Pathog..

[50] Ferreira A.R., Ramalho A.C., Marques M., Ribeiro D. (2020). The Interplay between Antiviral Signalling and Carcinogenesis in Human Papillomavirus Infections. Cancers.

[51] Scott M.L., Woodby B.L., Ulicny J., Raikhy G., Orr A.W., Songock W.K., Bodily J.M. (2020). Human Papillomavirus 16 E5 Inhibits Interferon Signaling and Supports Episomal Viral Maintenance. J. Virol..

[52] Guo H., König R., Deng M., Riess M., Mo J., Zhang L., Petrucelli A., Yoh S.M., Barefoot B., Samo M. (2016). NLRX1 Sequesters STING to Negatively Regulate the Interferon Response, Thereby Facilitating the Replication of HIV-1 and DNA Viruses. Cell Host Microbe.

[53] Lei Y., Wen H., Yu Y., Taxman D.J., Zhang L., Widman D.G., Swanson K.V., Wen K.W., Damania B., Moore C.B. (2012). The mitochondrial proteins NLRX1 and TUFM form a complex that regulates type I interferon and autophagy. Immunity.

[54] Lei Y., Wen H., Ting J.P. (2013). The NLR protein, NLRX1, and its partner, TUFM, reduce type I interferon, and enhance autophagy. Autophagy.

[55] Sunthamala N., Thierry F., Teissier S., Pientong C., Kongyingyoes B., Tangsiriwatthana T., Sangkomkamhang U., Ekalaksananan T. (2014). E2 proteins of high risk human papillomaviruses down-modulate STING and IFN-κ transcription in keratinocytes. PLoS ONE.

[56] Reiser J., Hurst J., Voges M., Krauss P., Münch P., Iftner T., Stubenrauch F. (2011). High-risk human papillomaviruses repress constitutive kappa interferon transcription via E6 to prevent pathogen recognition receptor and antiviral-gene expression. J. Virol..

[57] Park J.S., Kim E.J., Kwon H.J., Hwang E.S., Namkoong S.E., Um S.J. (2000). Inactivation of interferon regulatory factor-1 tumor suppressor protein by HPV E7 oncoprotein. Implication for the E7-mediated immune evasion mechanism in cervical carcinogenesis. J. Biol. Chem..

[58] Perea S.E., Massimi P., Banks L. (2000). Human papillomavirus type 16 E7 impairs the activation of the interferon regulatory factor-1. Int. J. Mol. Med..

[59] Karim R., Tummers B., Meyers C., Biryukov J.L., Alam S., Backendorf C., Jha V., Offringa R., van Ommen G.J., Melief C.J. (2013). Human papillomavirus (HPV) upregulates the cellular deubiquitinase UCHL1 to suppress the keratinocyte’s innate immune response. PLoS Pathog..

[60] He C., Lv X., Huang C., Angeletti P.C., Hua G., Dong J., Zhou J., Wang Z., Ma B., Chen X. (2019). A Human Papillomavirus-Independent Cervical Cancer Animal Model Reveals Unconventional Mechanisms of Cervical Carcinogenesis. Cell Rep..

[61] Wang X., Wang H.K., Li Y., Hafner M., Banerjee N.S., Tang S., Briskin D., Meyers C., Chow L.T., Xie X. (2014). microRNAs are biomarkers of oncogenic human papillomavirus infections. Proc. Natl. Acad. Sci. USA.

[62] Chiantore M.V., Mangino G., Iuliano M., Zangrillo M.S., De Lillis I., Vaccari G., Accardi R., Tommasino M., Fiorucci G., Romeo G. (2017). IFN-β antiproliferative effect and miRNA regulation in Human Papilloma Virus E6- and E7-transformed keratinocytes. Cytokine.

[63] Zhang J., Li S., Yan Q., Chen X., Yang Y., Liu X., Wan X. (2013). Interferon-β induced microRNA-129-5p down-regulates HPV-18 E6 and E7 viral gene expression by targeting SP1 in cervical cancer cells. PLoS ONE.

[64] He J., Ji Y., Li A., Zhang Q., Song W., Li Y., Huang H., Qian J., Zhai A., Yu X. (2014). MiR-122 directly inhibits human papillomavirus E6 gene and enhances interferon signaling through blocking suppressor of cytokine signaling 1 in SiHa cells. PLoS ONE.

[65] Lu H., Gu X. (2019). MicroRNA-221 inhibits human papillomavirus 16 E1-E2 mediated DNA replication through activating SOCS1/Type I IFN signaling pathway. Int. J. Clin. Exp. Pathol..

[66] Honegger A., Leitz J., Bulkescher J., Hoppe-Seyler K., Hoppe-Seyler F. (2013). Silencing of human papillomavirus (HPV) E6/E7 oncogene expression affects both the contents and the amounts of extracellular microvesicles released from HPV-positive cancer cells. Int. J. Cancer.

[67] Poiesz B.J., Ruscetti F.W., Gazdar A.F., Bunn P.A., Minna J.D., Gallo R.C. (1980). Detection and isolation of type C retrovirus particles from fresh and cultured lymphocytes of a patient with cutaneous T-cell lymphoma. Proc. Natl. Acad. Sci. USA.

[68] Yoshida M., Miyoshi I., Hinuma Y. (1982). Isolation and characterization of retrovirus from cell lines of human adult T-cell leukemia and its implication in the disease. Proc. Natl. Acad. Sci. USA.

[69] Gessain A., Barin F., Vernant J.C., Gout O., Maurs L., Calender A., de Thé G. (1985). Antibodies to human T-lymphotropic virus type-I in patients with tropical spastic paraparesis. Lancet.

[70] Osame M., Usuku K., Izumo S., Ijichi N., Amitani H., Igata A., Matsumoto M., Tara M. (1986). HTLV-I associated myelopathy, a new clinical entity. Lancet.

[71] Zhang L.L., Wei J.Y., Wang L., Huang S.L., Chen J.L. (2017). Human T-cell lymphotropic virus type 1 and its oncogenesis. Acta Pharm. Sin..

[72] Yuen C.K., Chan C.P., Fung S.Y., Wang P.H., Wong W.M., Tang H.V., Yuen K.S., Jin D.Y., Kok K.H. (2016). Suppression of Type I Interferon Production by Human T-Cell Leukemia Virus Type 1 Oncoprotein Tax through Inhibition of IRF3 Phosphorylation. J. Virol..

[73] Hyun J., Ramos J.C., Toomey N., Balachandran S., Lavorgna A., Harhaj E., Barber G.N. (2015). Oncogenic human T-cell lymphotropic virus type 1 tax suppression of primary innate immune signaling pathways. J. Virol..

[74] Charoenthongtrakul S., Zhou Q., Shembade N., Harhaj N.S., Harhaj E.W. (2011). Human T cell leukemia virus type 1 Tax inhibits innate antiviral signaling via NF-kappaB-dependent induction of SOCS1. J. Virol..

[75] Olière S., Hernandez E., Lézin A., Arguello M., Douville R., Nguyen T.L., Olindo S., Panelatti G., Kazanji M., Wilkinson P. (2010). HTLV-1 evades type I interferon antiviral signaling by inducing the suppressor of cytokine signaling 1 (SOCS1). PLoS Pathog..

[76] Zhang J., Yamada O., Kawagishi K., Araki H., Yamaoka S., Hattori T., Shimotohno K. (2008). Human T-cell leukemia virus type 1 Tax modulates interferon-alpha signal transduction through competitive usage of the coactivator CBP/p300. Virology.

[77] Narulla M.S., Alsairi A., Charmier L., Noonan S., Conroy D., Hall W.W., Sheehy N. (2017). Positive and Negative Regulation of Type I Interferons by the Human T Cell Leukemia Virus Antisense Protein HBZ. J. Virol..

[78] Sugata K., Satou Y., Yasunaga J., Hara H., Ohshima K., Utsunomiya A., Mitsuyama M., Matsuoka M. (2012). HTLV-1 bZIP factor impairs cell-mediated immunity by suppressing production of Th1 cytokines. Blood.

[79] Koralnik I.J., Lemp J.F., Gallo R.C., Franchini G. (1992). In vitro infection of human macrophages by human T-cell leukemia/lymphotropic virus type I (HTLV-I). AIDS Res. Hum. Retrovir..

[80] Macatonia S.E., Cruickshank J.K., Rudge P., Knight S.C. (1992). Dendritic cells from patients with tropical spastic paraparesis are infected with HTLV-1 and stimulate autologous lymphocyte proliferation. AIDS Res. Hum. Retrovir..

[81] Hishizawa M., Imada K., Kitawaki T., Ueda M., Kadowaki N., Uchiyama T. (2004). Depletion and impaired interferon-alpha-producing capacity of blood plasmacytoid dendritic cells in human T-cell leukaemia virus type I-infected individuals. Br. J. Haematol..

[82] Datta A., Sinha-Datta U., Dhillon N.K., Buch S., Nicot C. (2006). The HTLV-I p30 interferes with TLR4 signaling and modulates the release of pro- and anti-inflammatory cytokines from human macrophages. J. Biol. Chem..

[83] Fenizia C., Fiocchi M., Jones K., Parks R.W., Ceribelli M., Chevalier S.A., Edwards D., Ruscetti F., Pise-Masison C.A., Franchini G. (2014). Human T-cell leukemia/lymphoma virus type 1 p30, but not p12/p8, counteracts toll-like receptor 3 (TLR3) and TLR4 signaling in human monocytes and dendritic cells. J. Virol..

[84] Bellon M., Lepelletier Y., Hermine O., Nicot C. (2009). Deregulation of microRNA involved in hematopoiesis and the immune response in HTLV-I adult T-cell leukemia. Blood.

[85] Prado J.C.M., Monezi T.A., Amorim A.T., Lino V., Paladino A., Boccardo E. (2018). Human polyomaviruses and cancer: An overview. Clinics.

[86] Liu W., MacDonald M., You J. (2016). Merkel cell polyomavirus infection and Merkel cell carcinoma. Curr. Opin. Virol..

[87] Khalili K., White M.K. (2006). Human demyelinating disease and the polyomavirus JCV. Mult. Scler..

[88] Hirsch H.H., Steiger J. (2003). Polyomavirus BK. Lancet Infect. Dis..

[89] Lemos B., Nghiem P. (2007). Merkel cell carcinoma: More deaths but still no pathway to blame. J. Invest. Derm..

[90] Lunder E.J., Stern R.S. (1998). Merkel-cell carcinomas in patients treated with methoxsalen and ultraviolet A radiation. N. Engl. J. Med..

[91] Verma S., Ziegler K., Ananthula P., Co J.K., Frisque R.J., Yanagihara R., Nerurkar V.R. (2006). JC virus induces altered patterns of cellular gene expression: Interferon-inducible genes as major transcriptional targets. Virology.

[92] Co J.K., Verma S., Gurjav U., Sumibcay L., Nerurkar V.R. (2007). Interferon- alpha and-beta restrict polyomavirus JC replication in primary human fetal glial cells: Implications for progressive multifocal leukoencephalopathy therapy. J. Infect. Dis..

[93] De-Simone F.I., Sariyer R., Otalora Y.L., Yarandi S., Craigie M., Gordon J., Sariyer I.K. (2015). IFN-Gamma Inhibits JC Virus Replication in Glial Cells by Suppressing T-Antigen Expression. PLoS ONE.

[94] Abend J.R., Low J.A., Imperiale M.J. (2007). Inhibitory effect of gamma interferon on BK virus gene expression and replication. J. Virol..

[95] Willmes C., Adam C., Alb M., Völkert L., Houben R., Becker J.C., Schrama D. (2012). Type I and II IFNs inhibit Merkel cell carcinoma via modulation of the Merkel cell polyomavirus T antigens. Cancer Res..

[96] Liu W., Kim G.B., Krump N.A., Zhou Y., Riley J.L., You J. (2020). Selective reactivation of STING signaling to target Merkel cell carcinoma. Proc. Natl. Acad. Sci. USA.

[97] Dittmer D.P., Damania B. (2013). Kaposi sarcoma associated herpesvirus pathogenesis (KSHV)—An update. Curr. Opin. Virol..

[98] Hjalgrim H., Smedby K.E., Rostgaard K., Molin D., Hamilton-Dutoit S., Chang E.T., Ralfkiaer E., Sundström C., Adami H.O., Glimelius B. (2007). Infectious mononucleosis, childhood social environment, and risk of Hodgkin lymphoma. Cancer Res..

[99] Thorley-Lawson D.A., Hawkins J.B., Tracy S.I., Shapiro M. (2013). The pathogenesis of Epstein-Barr virus persistent infection. Curr. Opin. Virol..

[100] Gupta S., Ylä-Anttila P., Callegari S., Tsai M.H., Delecluse H.J., Masucci M.G. (2018). Herpesvirus deconjugases inhibit the IFN response by promoting TRIM25 autoubiquitination and functional inactivation of the RIG-I signalosome. PLoS Pathog..

[101] Gupta S., Ylä-Anttila P., Sandalova T., Sun R., Achour A., Masucci M.G. (2019). 14-3-3 scaffold proteins mediate the inactivation of trim25 and inhibition of the type I interferon response by herpesvirus deconjugases. PLoS Pathog..

[102] Gupta S., Ylä-Anttila P., Sandalova T., Achour A., Masucci M.G. (2020). Interaction With 14-3-3 Correlates With Inactivation of the RIG-I Signalosome by Herpesvirus Ubiquitin Deconjugases. Front. Immunol..

[103] Xu C., Sun L., Liu W., Duan Z. (2018). Latent Membrane Protein 1 of Epstein-Barr Virus Promotes RIG-I Degradation Mediated by Proteasome Pathway. Front. Immunol..

[104] Vilmen G., Glon D., Siracusano G., Lussignol M., Shao Z., Hernandez E., Perdiz D., Quignon F., Mouna L., Poüs C. (2021). BHRF1, a BCL2 viral homolog, disturbs mitochondrial dynamics and stimulates mitophagy to dampen type I IFN induction. Autophagy.

[105] Wang J.T., Doong S.L., Teng S.C., Lee C.P., Tsai C.H., Chen M.R. (2009). Epstein-Barr virus BGLF4 kinase suppresses the interferon regulatory factor 3 signaling pathway. J. Virol..

[106] Song Y.J., Izumi K.M., Shinners N.P., Gewurz B.E., Kieff E. (2008). IRF7 activation by Epstein-Barr virus latent membrane protein 1 requires localization at activation sites and TRAF6, but not TRAF2 or TRAF3. Proc. Natl. Acad. Sci. USA.

[107] Hahn A.M., Huye L.E., Ning S., Webster-Cyriaque J., Pagano J.S. (2005). Interferon regulatory factor 7 is negatively regulated by the Epstein-Barr virus immediate-early gene, BZLF-1. J. Virol..

[108] Wu L., Fossum E., Joo C.H., Inn K.S., Shin Y.C., Johannsen E., Hutt-Fletcher L.M., Hass J., Jung J.U. (2009). Epstein-Barr virus LF2: An antagonist to type I interferon. J. Virol..

[109] Geiger T.R., Martin J.M. (2006). The Epstein-Barr virus-encoded LMP-1 oncoprotein negatively affects Tyk2 phosphorylation and interferon signaling in human B cells. J. Virol..

[110] Liu X., Sadaoka T., Krogmann T., Cohen J.I. (2020). Epstein-Barr Virus (EBV) Tegument Protein BGLF2 Suppresses Type I Interferon Signaling To Promote EBV Reactivation. J. Virol..

[111] Shah K.M., Stewart S.E., Wei W., Woodman C.B., O’Neil J.D., Dawson C.W., Young L.S. (2009). The EBV-encoded latent membrane proteins, LMP2A and LMP2B, limit the actions of interferon by targeting interferon receptors for degradation. Oncogene.

[112] Michaud F., Coulombe F., Gaudreault E., Paquet-Bouchard C., Rola-Pleszczynski M., Gosselin J. (2010). Epstein-Barr virus interferes with the amplification of IFNalpha secretion by activating suppressor of cytokine signaling 3 in primary human monocytes. PLoS ONE.

[113] Chen Y., Fachko D.N., Ivanov N.S., Skalsky R.L. (2021). B Cell Receptor-Responsive miR-141 Enhances Epstein-Barr Virus Lytic Cycle via FOXO3 Inhibition. mSphere.

[114] Soltani S., Zakeri A., Tabibzadeh A., Zakeri A.M., Zandi M., Siavoshi S., Seifpour S., Farahani A. (2021). A review on EBV encoded and EBV-induced host microRNAs expression profile in different lymphoma types. Mol. Biol. Rep..

[115] Lu Y., Qin Z., Wang J., Zheng X., Lu J., Zhang X., Wei L., Peng Q., Zheng Y., Ou C. (2017). Epstein-Barr Virus miR-BART6-3p Inhibits the RIG-I Pathway. J. Innate Immun..

[116] Hooykaas M.J.G., van Gent M., Soppe J.A., Kruse E., Boer I.G.J., van Leenen D., Groot Koerkamp M.J.A., Holstege F.C.P., Ressing M.E., Wiertz E.J.H.J. (2017). EBV MicroRNA BART16 Suppresses Type I IFN Signaling. J. Immunol..

[117] Huang W.T., Lin C.W. (2014). EBV-encoded miR-BART20-5p and miR-BART8 inhibit the IFN-γ-STAT1 pathway associated with disease progression in nasal NK-cell lymphoma. Am. J. Pathol..

[118] Meckes D.G., Shair K.H., Marquitz A.R., Kung C.P., Edwards R.H., Raab-Traub N. (2010). Human tumor virus utilizes exosomes for intercellular communication. Proc. Natl. Acad. Sci. USA.

[119] Bray F., Ferlay J., Soerjomataram I., Siegel R.L., Torre L.A., Jemal A. (2018). Global cancer statistics 2018: GLOBOCAN estimates of incidence and mortality worldwide for 36 cancers in 185 countries. CA Cancer J. Clin..

[120] Tu T., Bühler S., Bartenschlager R. (2017). Chronic viral hepatitis and its association with liver cancer. Biol. Chem..

[121] Taylor P.E., Zuckerman A.J. (1968). Non-production of interfering substances by serum from patients with infectious hepatitis. J. Med. Microbiol..

[122] Blindenbacher A., Duong F.H., Hunziker L., Stutvoet S.T., Wang X., Terracciano L., Moradpour D., Blum H.E., Alonzi T., Tripodi M. (2003). Expression of hepatitis c virus proteins inhibits interferon alpha signaling in the liver of transgenic mice. Gastroenterology.

[123] Christen V., Duong F., Bernsmeier C., Sun D., Nassal M., Heim M.H. (2007). Inhibition of alpha interferon signaling by hepatitis B virus. J. Virol..

[124] Twu J.S., Lee C.H., Lin P.M., Schloemer R.H. (1988). Hepatitis B virus suppresses expression of human beta-interferon. Proc. Natl. Acad. Sci. USA.

[125] Tan G., Song H., Xu F., Cheng G. (2018). When Hepatitis B Virus Meets Interferons. Front. Microbiol..

[126] Duong F.H., Filipowicz M., Tripodi M., La Monica N., Heim M.H. (2004). Hepatitis C virus inhibits interferon signaling through up-regulation of protein phosphatase 2A. Gastroenterology.

[127] Tsunematsu S., Suda G., Yamasaki K., Kimura M., Izumi T., Umemura M., Ito J., Sato F., Nakai M., Sho T. (2017). Hepatitis B virus X protein impairs α-interferon signaling via up-regulation of suppressor of cytokine signaling 3 and protein phosphatase 2A. J. Med. Virol..

[128] Samal J., Kandpal M., Vivekanandan P. (2012). Molecular mechanisms underlying occult hepatitis B virus infection. Clin. Microbiol. Rev..

[129] Luangsay S., Gruffaz M., Isorce N., Testoni B., Michelet M., Faure-Dupuy S., Maadadi S., Ait-Goughoulte M., Parent R., Rivoire M. (2015). Early inhibition of hepatocyte innate responses by hepatitis B virus. J. Hepatol..

[130] Kawai T., Sato S., Ishii K.J., Coban C., Hemmi H., Yamamoto M., Terai K., Matsuda M., Inoue J., Uematsu S. (2004). Interferon-alpha induction through Toll-like receptors involves a direct interaction of IRF7 with MyD88 and TRAF6. Nat. Immunol..

[131] Liu S., Peng N., Xie J., Hao Q., Zhang M., Zhang Y., Xia Z., Xu G., Zhao F., Wang Q. (2015). Human hepatitis B virus surface and e antigens inhibit major vault protein signaling in interferon induction pathways. J. Hepatol..

[132] Wang J., Liu B., Wang N., Lee Y.M., Liu C., Li K. (2011). TRIM56 is a virus- and interferon-inducible E3 ubiquitin ligase that restricts pestivirus infection. J. Virol..

[133] Whitten T.M., Quets A.T., Schloemer R.H. (1991). Identification of the hepatitis B virus factor that inhibits expression of the beta interferon gene. J. Virol..

[134] Bai L., Zhang W., Tan L., Yang H., Ge M., Zhu C., Zhang R., Cao Y., Chen J., Luo Z. (2015). Hepatitis B virus hijacks CTHRC1 to evade host immunity and maintain replication. J. Mol. Cell Biol..

[135] Chen J., Xu W., Chen Y., Xie X., Zhang Y., Ma C., Yang Q., Han Y., Zhu C., Xiong Y. (2017). Matrix Metalloproteinase 9 Facilitates Hepatitis B Virus Replication through Binding with Type I Interferon (IFN) Receptor 1 To Repress IFN/JAK/STAT Signaling. J. Virol..

[136] Ciccaglione A.R., Stellacci E., Marcantonio C., Muto V., Equestre M., Marsili G., Rapicetta M., Battistini A. (2007). Repression of interferon regulatory factor 1 by hepatitis C virus core protein results in inhibition of antiviral and immunomodulatory genes. J. Virol..

[137] Taylor D.R., Shi S.T., Romano P.R., Barber G.N., Lai M.M. (1999). Inhibition of the interferon-inducible protein kinase PKR by HCV E2 protein. Science.

[138] Gale M.J., Korth M.J., Tang N.M., Tan S.L., Hopkins D.A., Dever T.E., Polyak S.J., Gretch D.R., Katze M.G. (1997). Evidence that hepatitis C virus resistance to interferon is mediated through repression of the PKR protein kinase by the nonstructural 5A protein. Virology.

[139] Taguchi T., Nagano-Fujii M., Akutsu M., Kadoya H., Ohgimoto S., Ishido S., Hotta H. (2004). Hepatitis C virus NS5A protein interacts with 2’,5’-oligoadenylate synthetase and inhibits antiviral activity of IFN in an IFN sensitivity-determining region-independent manner. J. Gen. Virol..

[140] Li X.D., Sun L., Seth R.B., Pineda G., Chen Z.J. (2005). Hepatitis C virus protease NS3/4A cleaves mitochondrial antiviral signaling protein off the mitochondria to evade innate immunity. Proc. Natl. Acad. Sci. USA.

[141] Li K., Foy E., Ferreon J.C., Nakamura M., Ferreon A.C., Ikeda M., Ray S.C., Gale M., Lemon S.M. (2005). Immune evasion by hepatitis C virus NS3/4A protease-mediated cleavage of the Toll-like receptor 3 adaptor protein TRIF. Proc. Natl. Acad. Sci. USA.

[142] Ding Q., Cao X., Lu J., Huang B., Liu Y.J., Kato N., Shu H.B., Zhong J. (2013). Hepatitis C virus NS4B blocks the interaction of STING and TBK1 to evade host innate immunity. J. Hepatol..

[143] Nitta S., Sakamoto N., Nakagawa M., Kakinuma S., Mishima K., Kusano-Kitazume A., Kiyohashi K., Murakawa M., Nishimura-Sakurai Y., Azuma S. (2013). Hepatitis C virus NS4B protein targets STING and abrogates RIG-I-mediated type I interferon-dependent innate immunity. Hepatology.

[144] Yu K., Li Q., Cheng Q., Huang C., Zheng J., Chen S., Ling Q., Zhu M., Chen M., Shi G. (2017). MicroRNA-548j inhibits type I interferon production by targeting ZBTB11 in patients with chronic hepatitis B. Biochem. Biophys. Res. Commun..

[145] Zhao Y., Yu Y., Ye L. (2019). MiR-3613-3p impairs IFN-induced immune response by targeting CMPK1 in chronic hepatitis B. Infect. Genet. Evol..

[146] Zhang L., Wang Z. (2020). Circular RNA hsa_circ_0004812 impairs IFN-induced immune response by sponging miR-1287-5p to regulate FSTL1 in chronic hepatitis B. Virol. J..

[147] Zhao X., Sun L., Mu T., Yi J., Ma C., Xie H., Liu M., Tang H. (2020). An HBV-encoded miRNA activates innate immunity to restrict HBV replication. J. Mol. Cell Biol..

[148] He C.L., Liu M., Tan Z.X., Hu Y.J., Zhang Q.Y., Kuang X.M., Kong W.L., Mao Q. (2018). Hepatitis C virus core protein-induced miR-93-5p up-regulation inhibits interferon signaling pathway by targeting IFNAR1. World J. Gastroenterol..

[149] Jarret A., McFarland A.P., Horner S.M., Kell A., Schwerk J., Hong M., Badil S., Joslyn R.C., Baker D.P., Carrington M. (2016). Hepatitis-C-virus-induced microRNAs dampen interferon-mediated antiviral signaling. Nat. Med..

[150] Mukherjee A., Di Bisceglie A.M., Ray R.B. (2015). Hepatitis C virus-mediated enhancement of microRNA miR-373 impairs the JAK/STAT signaling pathway. J. Virol..

[151] Gong W., Guo X., Zhang Y. (2018). Depletion of MicroRNA-373 Represses the Replication of Hepatitis C Virus via Activation of Type 1 Interferon Response by Targeting IRF5. Yonsei Med. J..

[152] Yan J., Zhang Y., Su Y., Tian L., Qin P., Xu X., Zhou Y. (2021). microRNA-125a targets MAVS and TRAF6 to modulate interferon signaling and promote HCV infection. Virus Res..

[153] Kondo Y., Kogure T., Ninomiya M., Fukuda R., Monma N., Ikeo K., Tanaka Y. (2019). The reduction of miR146b-5p in monocytes and T cells could contribute to the immunopathogenesis of hepatitis C virus infection. Sci. Rep..

